# Real-World Outcomes of DNA Damage Repair Altered Metastatic Castration-Resistant Prostate Cancer: Insights from FFPE-Based Genomic Profiling

**DOI:** 10.3390/cancers18152400

**Published:** 2026-07-25

**Authors:** Eleonora Lai, Francesco Pierantoni, Ilaria Zampiva, Davide Bimbatti, Melissa Ballestrin, Greta Pretto, Anna Milani, Elisa Erbetta, Salim Jubran, Chiara Pittarello, Andrea Di Marco, Nicolò Cavasin, Carolina Zamuner, Aichi Msaki, Lidia Moserle, Matteo Curtarello, Elisa Boldrin, Marco Montagna, Veronica Varano, Vasileios Mourmouras, Ivana Cataldo, Francesco Claps, Antonio Amodeo, Silvia Stragliotto, Marco Maruzzo, Umberto Basso

**Affiliations:** 1 Oncology Unit 3, Veneto Institute of Oncology IOV-IRCCS, 35128 Padua, Italy; francesco.pierantoni@iov.veneto.it (F.P.); melissa.ballestrin@iov.veneto.it (M.B.); greta.pretto@iov.veneto.it (G.P.); anna.milani@iov.veneto.it (A.M.); carolina.zamuner@iov.veneto.it (C.Z.); silvia.stragliotto@iov.veneto.it (S.S.); marco.maruzzo@iov.veneto.it (M.M.); 2 Oncology Unit 1, Veneto Institute of Oncology IOV-IRCCS, 35128 Padua, Italy; ilaria.zampiva@iov.veneto.it (I.Z.); davide.bimbatti@iov.veneto.it (D.B.); elisa.erbetta@asst-mantova.it (E.E.); salim.jubran@aulss2.veneto.it (S.J.); chiara.pittarello@iov.veneto.it (C.P.); andrea.dimarco@iov.veneto.it (A.D.M.); nicolo.cavasin@aulss2.veneto.it (N.C.); aichi.msaki@iov.veneto.it (A.M.); umberto.basso@iov.veneto.it (U.B.); 3 School of Specialization in Medical Oncology, Department of Oncology, University of Padua, 35128 Padua, Italy; 4 Oncology Unit, ASST Mantova-Carlo Poma Hospital, 46100 Mantova, Italy; 5 Medical Oncology Unit, Azienda ULSS 3 Serenissima, Chioggia Hospital, 30015 Chioggia, Italy; 6 Department of Medical Oncology, Azienda Unità Locale Socio-Sanitaria 2 Marca Trevigiana, 31100 Treviso, Italy; 7 Immunology and Molecular Oncology Diagnostics, Veneto Institute of Oncology IOV-IRCCS, 35128 Padua, Italy; lidia.moserle@iov.veneto.it (L.M.); matteo.curtarello@iov.veneto.it (M.C.); elisa.boldrin@iov.veneto.it (E.B.); marco.montagna@iov.veneto.it (M.M.); 8 Department of Pathology, Azienda Ospedaliera Universitaria Integrata di Verona, 37134 Verona, Italy; veronica.varano@aovr.veneto.it; 9 Anatomy and Pathological Histology, Veneto Institute of Oncology IOV-IRCCS, 35128 Padua, Italy; vasileios.mourmouras@iov.veneto.it (V.M.); ivana.cataldo@iov.veneto.it (I.C.); 10 Oncological Urology, Veneto Institute of Oncology IOV-IRCCS, 35128 Padua, Italy; francesco.claps@iov.veneto.it (F.C.); antonio.amodeo@iov.veneto.it (A.A.)

**Keywords:** metastatic castration-resistant prostate cancer (mCRPC), DNA Damage Repair (DDR), BRCA1, BRCA2, liquid biopsy, tissue genomic profiling, precision medicine

## Abstract

Background: Germline and somatic variants in DNA Damage Repair (DDR) genes are found in approximately one-fourth of patients with metastatic prostate cancer (PC). However, their precise prognostic role and predictive impact on standard therapies remain controversial. Methods: This retrospective, single-center study evaluated the prevalence of germline/somatic DDR aberrations in 287 eligible patients with metastatic prostate cancer (mPC), treated between 2017 and 2022. Clinical characteristics and treatment outcomes (PFS and OS) for chemotherapy (taxanes) or next-generation hormonal therapies (NHT) were compared between DDR-mutated (DDRmut) and wild-type (DDRwt) cohorts. Results: Sixty-three patients (21.9%) were DDRmut, with BRCA2 (12.5%), ATM (3.1%), and BRCA1 (1.39%) being the most common alterations. A family history of breast, ovarian, or prostate cancer strongly predicted DDRmut status (47.0% vs. 14.0%, *p* = 0.0001). Tissue samples remained evaluable for sequencing up to 180 months from collection. Overall baseline characteristics were similar between cohorts, and BRCA1/2- and ATM-mutated patients treated with first-line taxanes for mCRPC presented with non significantly highermedian OS compared to DDRwt patients (70 vs. 36 months; *p* = 0.30). On the contrary, the DDRmut subgroup showed a trend toward shorter PFS (12 vs. 18 months; *p* = 0.04) when treated with first-line NHT. No significant differences were observed with third-line Cabazitaxel. Conclusions: Formalin-fixed paraffin-embedded (FFPE) prostate tissue is highly reliable for DDR, possibly integrating novel liquid biopsy approaches for DDR evaluation. In a real-world setting, BRCA1/2 and ATM variants identify a distinct molecular subgroup that derives preferential survival benefit from first-line taxanes over standard hormonal intensification.

## Simple Summary

The retrospective, monocentric study evaluated the impact of DNA Damage Repair (DDR) gene variants in 287 patients with metastatic prostate cancer. Results showed that 21.9% of patients harbored DDR alterations (somatic or germline), with BRCA2, ATM, and BRCA1 being the most common. A positive family history of BRCA-related cancers was a strong predictor for a positive test for these variants. The analysis demonstrated that formalin-fixed paraffin-embedded (FFPE) prostate tissue samples can be successfully processed for genetic testing even after long storage periods of up to 180 months (15 years). BRCA1/2 or ATM alteration carriers showed a significant benefit from first-line taxane chemotherapy, with a longer overall survival compared to wild-type patients (70 vs. 36 months). On the contrary, novel hormonal therapies (NHT) showed a trend toward lower efficacy in this mutated subgroup.

## 1. Introduction

Molecular profiling has become an integrated part of personalized cancer therapy, with somatic genetic alterations offering potential targets for precision treatment strategies [1]. In prostate cancer, disruptions in the DNA Damage Repair (DDR) pathway, particularly involving BRCA1 and BRCA2, are the most therapeutically relevant genomic events [1,2]. Somatic DDR alterations are more prevalent than the germline ones, occurring in approximately 19–31% of advanced prostate cancer cases vs. 7.4–16.2%, respectively [1,2,3]. Germline BRCA1/2 variants are associated with aggressive disease phenotypes, including early metastasis and reduced overall survival, while the prognostic and predictive values of other somatic DDR alterations remain uncertain [1,3]. During recent times, liquid biopsy has emerged as a valuable tool for genomic profiling in metastatic prostate cancer because of its minimally invasive nature and the ability to capture newly acquired DDR variants as a possible result of tumor heterogeneity and colonial treatment pressure [4,5]; however, tissue-based genomic testing using formalin-fixed paraffin-embedded (FFPE) specimens is still fundamental for molecular diagnostics. Archival FFPE tissue is frequently available in routine clinical practice, provides high-quality DNA for comprehensive genomic profiling, and is particularly valuable when circulating tumor DNA is insufficient or unavailable [6].

Evidence suggests that metastatic castration-resistant prostate cancer (mCRPC) patients harboring DDR alterations benefit from poly(ADP-ribose) polymerase (PARP) inhibitors; however, their response to standard therapies such as taxanes and next-generation hormonal therapies (NHT) is less clearly defined [7,8]. In the past two years, there has also been a shift in the standard of care for the treatment of prostate cancer in the castration-sensitive setting. Currently, the therapeutic standard for high-volume patients, according to the main international guidelines, consists of a triplet therapy regimen including LHRH analogs, Docetaxel, and an NHT [9]. As a result, the vast majority of patients will no longer be NHT-naive at the time of progression to castration-resistant disease. Existing studies report conflicting outcomes regarding the associations between somatic DDR variants and clinical benefits from taxanes or NHT in castration-resistant settings. For example, Annala et al. have identified significantly shorter progression times in patients with BRCA2 or ATM variants treated with first-line mCRPC NHT, while other retrospective analyses suggest diminished PSA responses to docetaxel in BRCA2-mutant mCRPC [2,7,8]. All of these studies were conducted before the change in the therapeutic standard. Nonetheless, if we consider the epidemiological impact of prostate neoplasms (the second-most common cancer in men over 70 years old) and the efficacy of the treatment regimens that previously constituted the standard of care, we should expect that, in the coming years, a significant proportion of patients will reach the castration-resistant stage, potentially not being naïve to NHT. Given this change in perspective, this study aims to evaluate the clinical outcomes of mCRPC patients treated with taxanes and/or NHT, stratified by somatic DDR mutational status, as determined by a genomic assay.

## 2. Materials and Methods

We conducted a single-center retrospective analysis on all consecutive patients with mPC who were treated at the Veneto Institute of Oncology between 2017 and 2022, and for whom DDR genetic test result was available. The eligibility criteria were: age ≥ 18 years; confirmed histological diagnosis of prostate cancer; metastatic disease (extrapelvic lymph nodes or distant metastases, any site) detected with any imaging (CT scan, MRI, bone scintigraphy, and Choline or PSMA PET/CT); molecular analyses of DDR variants assessed using two commercially available tests depending on the year and clinical availability (Foundation ONE -Foundation Medicine, Inc., Cambridge, MA, USA- and Devyser NGS kit CE-IVD - Devyser AB, Stockholm, Sweden), the latter focused only on BRCA1 and BRCA2 genes); and signed informed consent. Informed consent was obtained from all subjects involved in the study. Two patients’ cohorts were defined: those with DDR gene mutations (DDRmut), identified at the germline or somatic level, and those without such alterations (DDRwt). Variants were classified according to ENIGMA BRCA1- and BRCA2-specific guidelines and to the criteria adopted by the commercially available assays used for genomic profiling. Clinical data were retrospectively extracted from electronic medical records, including age at diagnosis, PSA levels, family history of cancer, Gleason score, histologic variants, tumor stage, metastatic status and sites, as well as details on systemic therapies, treatment response, and survival outcomes. The study received institutional review board approval on 31 March 2022, and was conducted in accordance with the Declaration of Helsinki and Good Clinical Practice Guidelines.

Our primary endpoint was to compare progression-free survival (PFS) between patients with mutated vs. wild-type DDR status (DDRmut vs. DDRwt) across different systemic treatments in the castration-resistant phase. Variants of uncertain significance (VUS) were categorized within the DDR-mutated subgroup for descriptive molecular characterization; however, they were excluded from survival analyses due to the uncertain pathogenic and functional significance of these alterations. Subsequently, predefined subgroup analyses focused on BRCA1/2 and ATM mutations, given their established therapeutic relevance. PFS was measured from the start of a new therapy to radiographic/clinical progression or death, whichever occurred first. Disease progression was assessed via imaging, using Response Evaluation Criteria in Solid Tumors (RECIST) v.1.1 for soft tissue/visceral lesions and modified Prostate Cancer Working Group 3 criteria for bone metastases. Data were analyzed by drug class, treatment line, and disease stage for patients who received standard treatments for mCRPC and for whom complete follow up data were available. Three clinical scenarios were consequently identified as follows:

- First-line NHT for mCRPC;
- First-line Taxanes for mCRPC;
- Subsequent therapies for patients previously exposed to one ARSi and Docetaxel.

The secondary endpoints were to compare clinical characteristics between the two different subgroups (age, histology, PSA and stage at diagnosis, and disease volume), compare the frequency of prostate, breast, and ovarian cancer between the family histories of DDRmut patients and DDRwt patients, to compare the duration of the castration-sensitive phase of DDRmut and DDRwt patients, calculated from the start of androgen-deprivation therapy to the onset of the mCRPC phase, and to compare overall survival (OS) in the mCRPC phase of DDRmut and DDRwt patients, calculated as the time from the commencement of systemic therapy for mCSPC and mCRPC until death (any cause). Results were reported using descriptive statistics and compared using the appropriate tests (Chi-square, Fishers’ exact test, t-Student, and logistic regression analysis). Time-to-event outcomes (PFS and OS) were analyzed using the Kaplan–Meier method, and medians are reported with 95% CI. The log rank test was used to compare patient subgroup outcomes. Statistical analyses were performed using “R” software v.4.5.1 (R Foundation for Statistical Computing, Vienna, Austria).

## 3. Results

### 3.1. Patient Selection and Main Clinical Features

A total of 331 mPC patients underwent genetic testing for DDR variants. A genomic assay on prostate tumor specimens was performed on all patients. Forty-four DNA samples were “non-evaluable” or “inadequate”. Therefore, a total of 287 patients were eligible for the present analysis.

**Figure 1:**
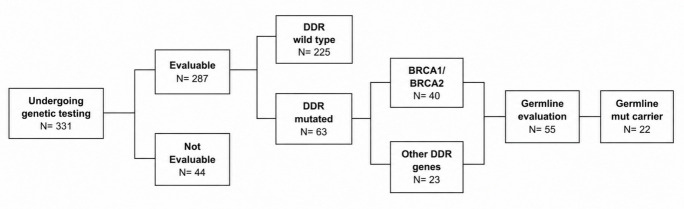
Total numbers for genetic testing.

Over half of our sample underwent genomic testing on both prostate cancer tissue and circulating tumor DNA (196–59.22%). Twenty-one percent (21%) of patients had a relevant mutation in DDR genes (DDRmut) in tumor tissue, while 78.4% of patients were found to be wild type (DDRwt), as reported in Figure 1.

Among the 63 DDRmut patients, 55 underwent germline evaluation. We then found that 22/55 were germline carriers of DDR alterations.

The main clinical features of the eligible population are reported in Table 1. We did not observe any differences in baseline clinical or pathologic characteristics between DDRmut and DDRwt patients.

In our sample, a positive familiar history for typically BRCA-related tumors (such as pancreatic/ovarian/breast cancer), and in particular for ovarian cancer, was a strong predictor for a DDRmut result (*p*-value < 0.0001), as shown in Table 2. Family history was investigated within the oncological visits, and always prior to proposing BRCA1/2 testing; however, this was for informational purposes only and not to determine eligibility for DDR assessment.

### 3.2. Types of Variants

Sixty-three patients (22%) had at least one DDR alteration. The most frequently identified variants regarded BRCA2 gene (36/287 patients, 12.5%), followed by ATM (9/287 patients, 3.1%), and BRCA1 (4/287 patients, 1.39%). The subgroup of BRCA1/2 and ATM patients represented 16.3% of patients and 74% of the DDRmut patients. Two patients were found to harbor both BRCA2 and ATM germline variants. Two other patients carried both somatic BRCA2 and PIK3CA, PTEN and TP53 mutations. Other DDR alterations were CHEK2 (6 patients), PALB2 (3 patients), FANCA (2 patients), CDK12 (2 patients), and BRIP1A (1 patient). The pathogenic alterations were highly heterogeneous, with a great proportion of frameshift insertions or deletions, and nonsense/stop mutations. Among the identified alterations, 6 were variants of uncertain significance (VUS): 3 BRCA2 VUS, 2 FANCA VUS, and 1 CHEK2 VUS. Detailed numbers of somatic and germline variants of each gene are outlined and a complete list of all DDR aberrations is included in Table A1.

### 3.3. Interval from Surgery/Biopsy to Genetic Testing

The median time from prostatectomy or prostate biopsy to genetic testing in the whole population was 38 months (range 1–258 months). For one patient, we could perform genomic profiling after 180 months (about 15 years). Prostate tumor specimens of 44 patients did not yield adequate DNA for genomic assay. These samples had a median time from diagnosis to analysis of 77.50 months (range 37–258), significantly higher compared to the median time of 30.50 months (range 1–190) of samples with adequate yield of DNA, *p*-value = 0.002. The most common reason for “not evaluable” samples was the missed minimum quality control metrics for laboratory processing (31 out of 44, 70%), followed by failure to meet minimum requirements for amount of extracted DNA (8 out of 44, 18.18%). Three cases presented with a problem of low exon coverage, and two cases with issues in both quality and quantity of extracted DNA.

### 3.4. Time to Castration Resistance

DDRmut patients showed a trend for shorter duration of the castration sensitivity compared to DDRwt cohort (36 vs. 44 months, *p*-value = 0.81, HR 0.71, 95% CI: 0.38–1.01). When compared to DDRwt patients, ATM/BRCA1/2mut patients presented with a significantly shorter median duration of castrate sensitive phase of 25 months, with *p*-value = 0.003 (HR = 0.49, 95% CI: 0.26–0.95), as shown in Table 3.

### 3.5. PFS and OS for Taxanes as First-Line Treatment for mCRPC

**Figure 2:**
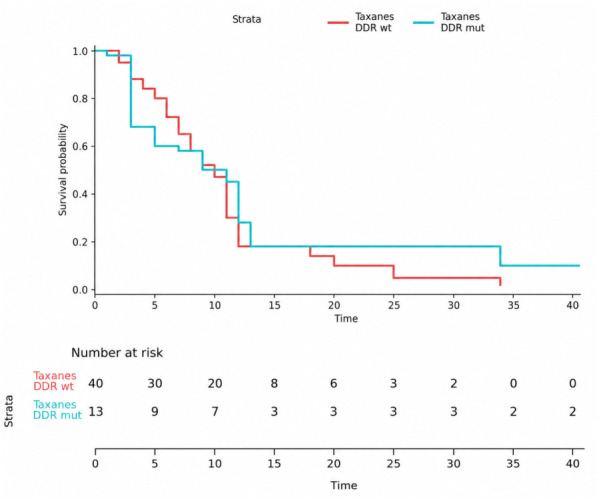
PFS for taxanes as first-line treatment in mCRPC pts among DDR wt and DDRmut pts.

Among the 53 patients mCRPC treated with first-line taxanes, PFS was of 10 months for DDRwt pts (40 pts) and 9.5 months for DDRmut pts (13pts) (*p*-value = 0.85, HR 1.79, 95% CI 0.84–3.83), with no significant difference, as shown in Figure 2.

**Figure 3:**
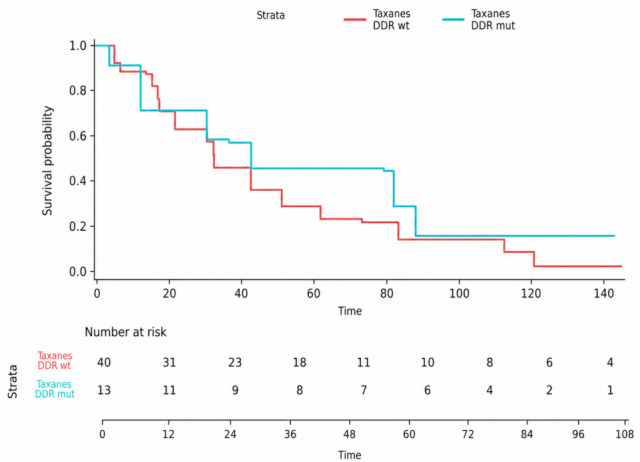
OS for taxanes as first-line treatment in mCRPC pts among DDR wt and DDRmut pts.

Similarly, no statistical differences were found between the two cohorts in terms of OS for the same treatment, as reported in Figure 3.

Nonetheless, DDRmut patients showed a trend towards a longer median OS when compared with DDRwt patients (39 vs. 36 months, *p*-value = 0.58, HR 0.84, 95% CI 0.415–1.29).

### 3.6. PFS and OS for NHT as First-Line Treatment for mCRPC

**Figure 4:**
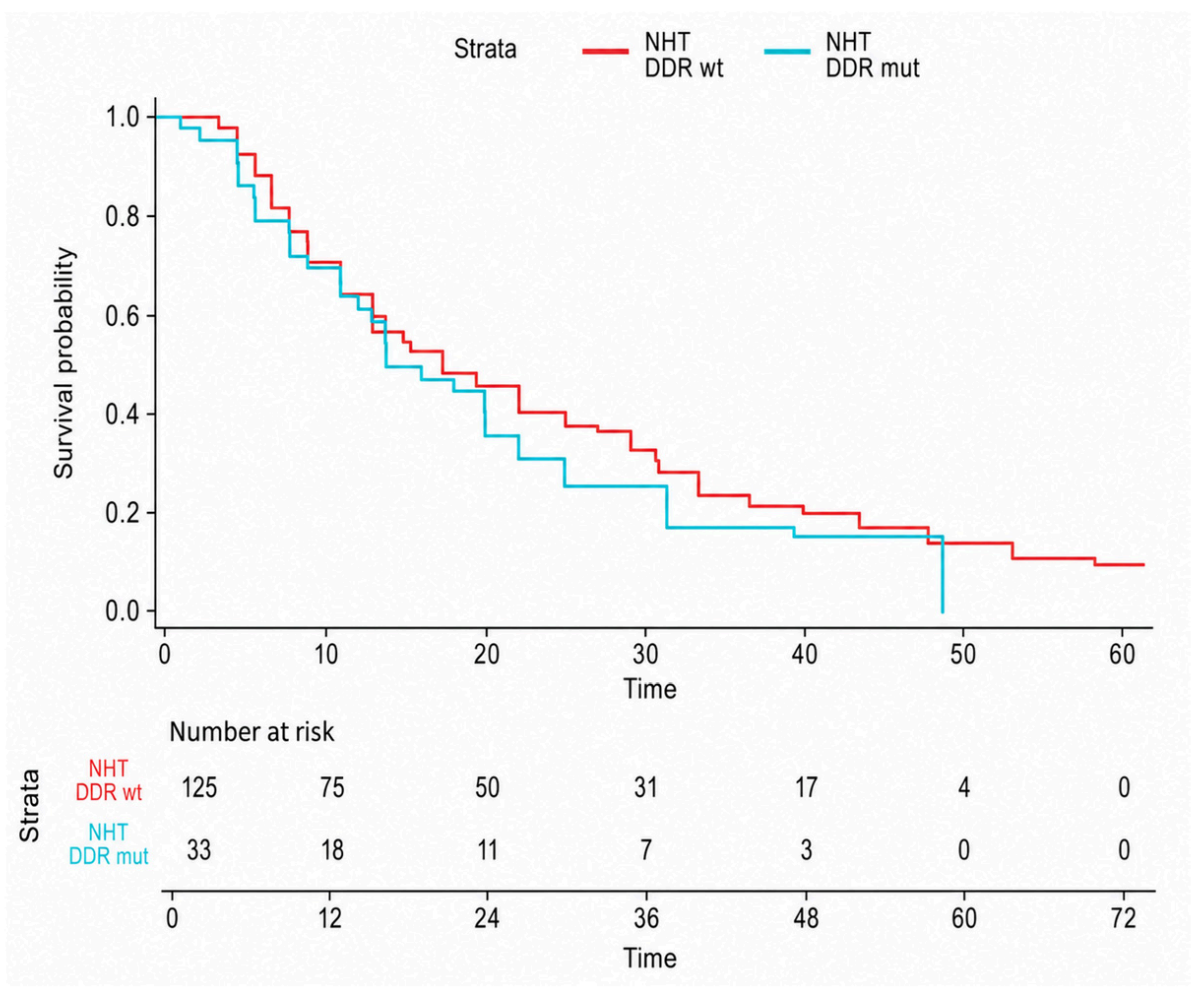
PFS for NHT as first-line treatment in mCRP pts among DDR wt and DDRmut pts.

Among the 158 patients treated in first line with NHTs, no differences were found in PFS between DDRwt and DDRmut patients (18 vs. 17 months, *p*-value = 0.3, HR 0.88, 95% CI 0.499–1.201), as reported in Figure 4.

**Figure 5:**
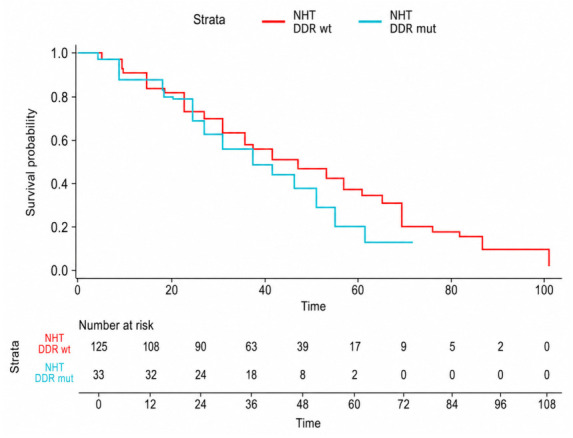
OS for NHT as first-line treatment in mCRPC pts among DDR wt and DDRmut pts.

Median OS was of 44 months for DDRwt pts (125) and of 40 months for DDRmut pts (33) (*p*-value = 0.89, HR 0.97, 95% CI 0.24–1.016), again with no significant difference, as reported in Figure 5.

**Figure 6:**
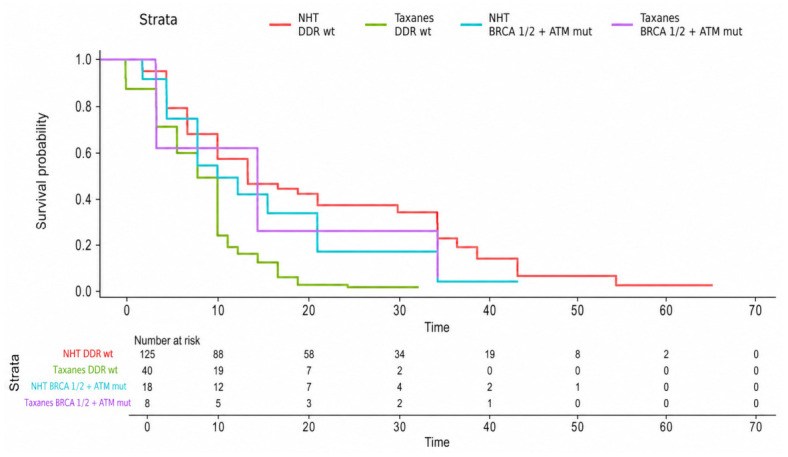
PFS for NHT and taxanes as first-line treatment in mCRPC pts among DDR wt and BRCA 1/2 + ATM mut pts.

**Figure 7:**
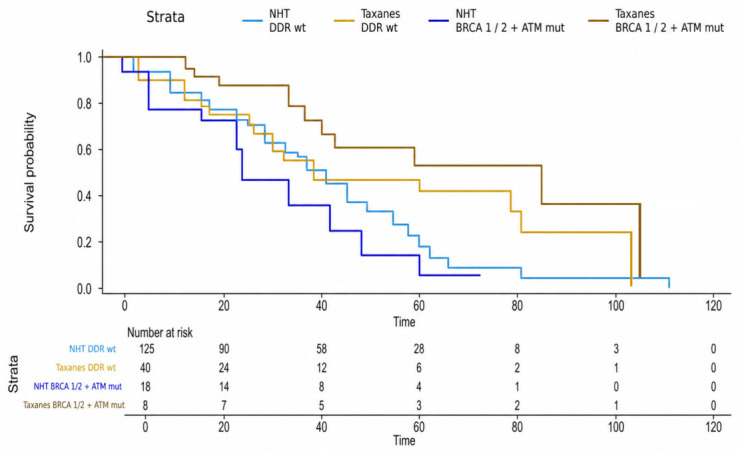
OS for NHT and taxanes as first-line treatment in mCRPC pts among DDR wt and BRCA 1/2 + ATM mut pts.

Given the large sample size of DDR-mutated patients in this disease setting, we decided to evaluate PFS and OS in four different patient cohorts, as reported respectively in Figure 6 and Figure 7, and classified as follows: DDRwt patients treated with NHT; DDRwt patients treated with taxanes; BRCA1/2- and/or ATM-mutated patients treated with NHT; BRCA1/2- and/or ATM-mutated patients treated with taxanes. In this setting, 18 patients with BRCA1/2 mut and/or ATM mutations were treated with NHT: their median PFS was of 12 months, and the median OS was of 38 months; DDRwt patients showed a trend towards better outcomes, with a median PFS of 18 months (*p*-value = 0.04, HR 0.747, 95% CI 0.396–0.754), and a median OS of 44 months (*p*-value = 0.37, HR 0.98, 95% CI 0.38–1.005).

In the same setting, eight patients with BRCA1/2 and ATM alterations received taxanes as a first-line MCRPC treatment. We found no significant differences between these two subgroups: the mutated patients showed a median PFS of 12 months, and a median OS of 70 months, while DDRwt patients had a median PFS of 10 months (*p*-value = 0.50, HR 0.88, 95% CI 0.413–1.045) and a median OS of 36 months (*p*-value = 0.30, HR = 0.64, 94% CI 0.38–1.90).

### 3.7. Multivariable Cox Model

Given the retrospective nature of our dataset, and the consequently derived heterogeneity, we performed multivariable Cox proportional hazards regression analyses for the main survival endpoints, including first-line mCRPC PFS and OS. The models were adjusted for clinically relevant covariates, which were selected based on their established prognostic relevance, and also on data availability, including age at diagnosis, Gleason score, metastatic burden, disease presentation (de novo vs. metachronous metastatic disease), and previous systemic treatments. Cox model showed that DDRmut status was not independently associated with worse PFS (*p*-value = 0.669, HR 1.08, 95% CI 0.76–1.53) or OS (*p*-value = 0.647, HR 1.14, 95% CI 0.65–2.02) in first-line treatment for mCRPC. The results are summarized in Table 4.

### 3.8. PFS and OS for Cabazitaxel as Third-Line Treatment for mCRPC

**Figure 8:**
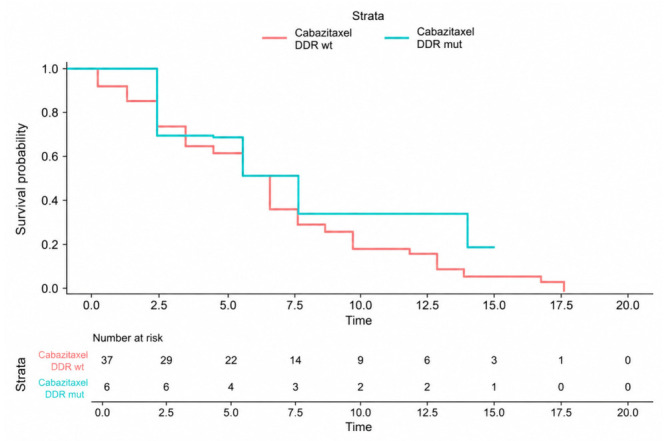
PFS for Cabazitaxel as third-line treatment in mCRPC pts among DDR wt and DDRmut pts.

Forty-three patients progressed to both one taxane-based line and one NHT treatment, and received Cabazitaxel as a third-line treatment. Among them, 37 were DDRwt and 6 were DDRmut. Median PFS for this line of treatment was 6 months for DDRwt patients and 7 months for DDRmut patients (*p*-value = 0.34, HR 0.99, 95% CI, 0.721–1.03), as reported in Figure 8.

**Figure 9:**
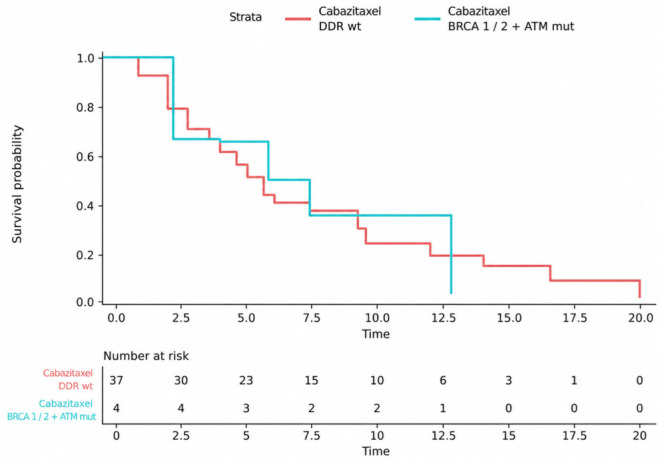
PFS for Cabazitaxel as third-line treatment in mCRPC pts among DDRwt and BRCA 1/2 + ATM mut pts.

The subcohort of BRCA1/2 mut and/or ATM mut patients (4 pts) performed similarly, as shown in Figure 9, with a median PFS of 6 months, with no significant difference when compared to DDRwt patients (*p*-value = 0.86, HR 0.86, 95% CI, 0.654–1.201).

**Figure 10:**
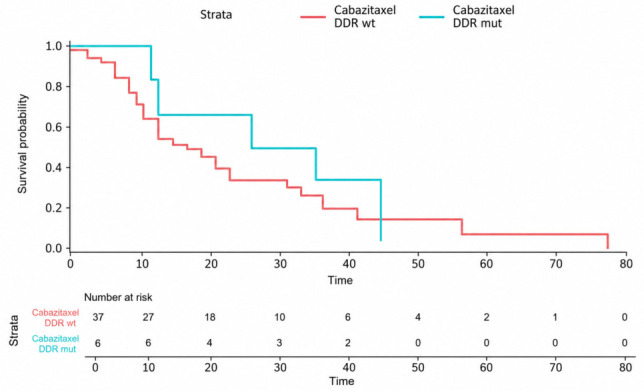
OS for Cabazitaxel as third-line treatment in mCRPC pts among DDR wt and DDRmut pts.

In the same setting, we observed a median OS of 17 months for DDRwt cohort and of 29.5 months for DDRmut pts, with no statistical significance (*p*-value = 0.23, HR 0.94, 95% CI, 0.534–1.112), as reported in Figure 10.

**Figure 11:**
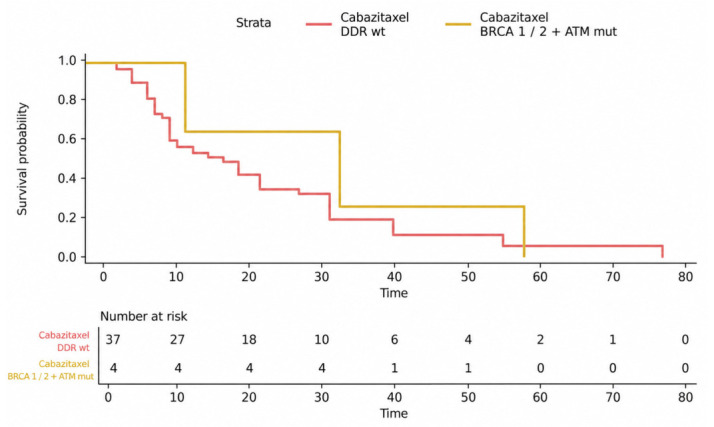
OS for Cabazitaxel as third-line treatment in mCRPC pts among DDR wt and BRCA 1/2 + ATM mut pts.

The subcohort of BRCA1/2 mut and ATM mut patients (4 pts) performed slightly better, with a median OS of 31.3 months, yet with no significant difference when compared to DDRwt patients (*p*-value = 0.54, HR 0.99, 95% CI, 0.698–1.097), as shown in Figure 11.

Complete results with HR, Cis, and log ranks providing comparisons between different treatment lines are reported in Table 5 and Table 6. Hazard ratios were calculated using the BRCA1/2-ATM or DDRmut groups as the reference category.

## 4. Discussion

In this real-world cohort, 21% of patients diagnosed with mCRPC harbored alterations in DDR genes. Variants specifically involving BRCA1/2 and ATM accounted for 15% of the cohort: this data is not only consistent with previously published data such as PROfound and TRITON trial [2,10], but it also reflects the central role of BRCA1/2 alterations among clinically actionable DDR alterations [2].

Clinical and pathological features did not significantly differ between patients with DDR variants (DDRmut) and those with wild-type DDR genes (DDRwt); however, the presence of DDR alterations was associated with a shorter duration of castration sensitivity. Although this observation did not reach statistical significance, it is in line with existing evidence that suggest a more aggressive biological behavior in tumors harboring DDR alterations, particularly BRCA2 [2,7]. Furthermore, in our cohort, a positive family history of malignancies typically associated with BRCA alterations (such as ovarian and/or breast cancer) showed a strong correlation with the presence of DDR gene alterations, extending beyond BRCA1/2 and ATM. In our cohort, ovarian cancer family history showed the strongest association with DDR alterations, likely reflecting its high specificity for hereditary BRCA-associated syndromes. On the other hand, prostate cancer is highly prevalent in the general population, particularly among older men, reducing its specificity as an indicator of hereditary cancer predisposition. Similarly, the relatively low number of breast cancer cases reported in our cohort may have limited the statistical power to detect a significant association. However, several studies have demonstrated that appropriate oncogenetic counseling can enhance the detection rate of BRCA1 and BRCA2 mutations, while also mitigating the psychological burden of diagnosis and supporting preventive care strategies for at-risk relatives [11]. Furthermore, considering the actual treatment landscape, current European and Italian oncological guidelines recommend somatic testing for BRCA1/2 variants in all patients with metastatic prostate cancer, in order to obtain not only purely prognostic information, but also to guide treatment selection for PARP-inhibitor-based strategies [9,11,12]. However, considering the variability in access to genetic testing and the extended timeframes often required for analysis, a detailed and systematic collection of family history may serve as a useful tool to prioritize patients who are more likely to benefit from timely molecular testing [12].

With regard to the detection efficiency of DDR gene alterations, our dataset demonstrated a 14% assay failure rate, which is notably lower than other data commonly reported in the literature [10]. Failure rate in assessing DDR variants is particularly relevant in prostate cancer, where archival tissue samples frequently exceed five years in age at the time of molecular analysis, potentially compromising nucleic acid integrity and test performance. The risk of nucleic acid degradation increases with the duration of storage of histological specimens in FFPE blocks. In bone biopsies, sample degradation may occur at even earlier stages, particularly during the decalcification process [12,13]. Consequently, optimal sample preparation is essential and should be coordinated with the genetic testing laboratory to ensure specimen quality. To maximize the success rate of genetic analyses, certain European countries have implemented guidelines that exclude prostate cancer histological samples older than 10 years from genetic testing [13].

When somatic assay is not available or evaluable, characterizing the circulating tumor DNA (ctDNA) percentage in circulating cell-free DNA (cfDNA) may fulfill the need for a non-invasive assay that can be repeated to assess treatment response and resistance at the time of disease progression. In addition, ctDNA profiling is not confounded by the sampling bias of a biopsy of a single metastatic lesion and may capture tumor heterogeneity. Still, liquid biopsy faces technical and interpretational issues: alterations found in FFPE may not be highlighted in liquid biopsy due to low ctDNA concentrations, leading to false negatives [6,14]. However, more recently artificial intelligence (AI)-based analytical approaches are increasingly being integrated with circulating tumor DNA (ctDNA) profiling to improve variant interpretation, identify complex genomic patterns, and support individualized treatment selection [15]. Although these technologies are expected to enhance the sensitivity and clinical utility of liquid biopsy, they currently complement rather than replace tissue-based genomic profiling. FFPE-derived DNA continues to represent a robust and widely available source for comprehensive molecular characterization, particularly when ctDNA yield is low or when archival tumor tissue is readily accessible. Therefore, the integration of tissue- and liquid-based molecular testing, supported by advanced computational approaches, is likely to represent the future direction of precision oncology in prostate cancer [16].

Within our cohort, histological samples up to 15 years old were successfully analyzed, with more recent specimens (<5 years) demonstrating a lower failure rate, albeit without reaching statistical significance. Recent data highlight the need for timely testing for FFPE samples, ideally with a cut off of 36 months of storage duration [14]. In that series samples older than 36 months showed lower levels of DNA concentrations and higher success rate for variants identification [14]. It would be consequently prudent to prioritize the analysis of samples at the earliest opportunity, while minimizing the use of bone biopsies when possible [16].

However, adequate genomic profiling represents only the first step toward its clinical application, because the therapeutic relevance of DDR alterations varies considerably according to the gene involved. As previously stated, the decision to separately evaluate BRCA1/2 and ATM alterations reflects the already established biological heterogeneity of DDR genes; also, the same decision was made to ensure comparability with pivotal clinical trials reported in the literature, such as the PROfound trial [5], while recognizing the small identified subgroups, the retrospective nature of this study and the consequently exploratory nature of these findings.

This subgroups division revealed trends for different outcomes between BRCA1/BRCA2/ATM mut and wild-type groups, but without any statistical significance.. These findings, which will be elaborated upon subsequently, suggest that alterations in “non-BRCA1/2-ATM” DDR genes may confer distinct predictive and prognostic significance. This distinction was highlighted in the PROfound trial, where the therapeutic benefit of PARP inhibitor monotherapy was predominantly observed in patients with BRCA1/2 and ATM variants [17]. It is important to acknowledge that prostate cancer, especially in the castration-resistant setting, exhibits marked intratumoral heterogeneity and clonal diversification. Preclinical evidence supports the potential efficacy of immunotherapeutic agents and ATR inhibitors in specific subsets of “non-BRCA1/2-ATM” DDR-mutated tumors [18]. Further studies are warranted to explore these aspects and also to improve molecular stratification beyond the current HRR classification.

With regard to therapeutic response across different lines of treatment, our cohort exhibited a statistically significant difference in overall survival (OS) during first-line mCRPC therapy between patients harboring BRCA1/2-ATM mutations and those with DDRwt status. Notably, BRCA1/2/ATM mut patients showed a trend towards improved clinical outcomes when treated with taxanes in the first-line mCRPC setting. Conversely, this subgroup exhibited a non-significant trend toward inferior outcomes (both OS and PFS) when treated with NHT as first-line treatment compared to DDRwt patients. Furthermore, in the third-line mCRPC setting, we found a trend for the improved efficacy of Cabazitaxel in patients with BRCA1/2/ATM variants compared to the DDRwt counterparts. Although these exploratory analyses generated clinically relevant hypotheses regarding potential differential treatment sensitivity, the limited sample size (particularly for patients harboring BRCA1/2 or ATM alterations) reduced statistical power and increased the possibility of both false-positive and false-negative findings. Consequently, these observations should not be considered definitive and warrant validation in larger prospective biomarker-driven studies. 

Our study has also other limitations. The data collection was monocentric and retrospective, including a heterogeneous patients’ cohort evaluated for DDR variants at various disease stages and using different assay platforms. In particular, in 34/331 patients, the DDR assessment only evaluated BRCA1 and BRCA2 alterations, leaving aside other DDR variants; however, since the clinical and prognostic implications of non-BRCA1/2 mutations are less clear, and also considering the reduced number of patients tested with the Devyser kit, the impact of this heterogeneity is likely limited; also, BRCA1/2 variants were consistently evaluated across the testing platforms, limiting the impact on the principal analyses. However, the use of two different commercially available assays with variable gene coverage actually reflects the progressive implementation of molecular diagnostics in routine clinical practice. Another limitation regarding germline mutation evaluation: we identified this kind of pathogenic variant in a subset of patients, but the limited number of germline carriers precluded a reliable comparison between germline and somatic alterations. Future prospective studies with standardized comprehensive genomic profiling and adequately powered molecular subgroups will be essential to clarify the respective prognostic and predictive roles of germline and somatic DDR alterations.

Additionally, within the first-line mCRPC setting, patients treated with taxanes predominantly had prior exposure to novel hormonal therapies (NHT) during the metastatic castration-sensitive prostate cancer (mCSPC) phase. Conversely, patients receiving NHT in first-line mCRPC more frequently had preceding docetaxel (plus NHT in some cases) treatment in the mCSPC setting. Such treatment sequencing may have introduced selection biases, resulting in biologically distinct subpopulations and potentially explaining the observed disparities in treatment outcomes.

Furthermore, the multivariable Cox regression analyses did not identify DDR status as an independent predictor of progression-free or overall survival after adjustment for relevant clinical covariates, further supporting a cautious interpretation of the subgroup findings.

However, when focusing on first-line treatment for mCRPC setting, the literature reports conflicting data; our study is in line with previous data reported by Annala et al. [8]. They analyzed the retrospective data of 176 patients with mCRPC, including 22 gDDR carriers (BRCA2; *n* = 16), and found that the PFS of gDDR carriers on first-line NHT for mCRPC was significantly shorter than that of noncarriers (3.3 vs. 6.2 months; *p* = 0.01) [8]. This data was successively confirmed in PROREPAIR-B, which explored the predictive impact of BRCA1/2 and ATM alterations identified in circulating tumor DNA [19]. On the contrary, Antonarakis et al. reported a trend toward a more prolonged PFS in gDDR carriers (13.3 vs. 10.3 months; *p* = 0.107) and ATM/BRCA1/BRCA2 carriers (15 vs. 10.8 months; *p* = 0.090) compared with noncarriers [20]. However, in this latter study, many patients had previously received carboplatin chemotherapy, which could have biased the final result.

In relation to the outcomes in third-line treatment, a retrospective series found no significant differences for treatment with Cabazitaxel, neither in terms of PSA responses nor mPFS between DDRwt and DDRmut patients (mPFS of 5.95 vs. 6.57 months, *p*-value = 0.55, respectively) [21]. No difference was found in the median OS between DDRmut and DDRwt groups (12.9 months vs. 12.5 months, *p*-value = 0.19).

In the same retrospective series, 10 DDR-mutated patients received treatment with PARP-inhibitors before Cabazitaxel: none showed PSA response, and all were associated with shorter mPFS (3.08 months) and mOS (3.08 months), yet with no statistical significance. The efficacy of Cabazitaxel in patients with DDR mutational status may be lower in men previously treated with PARP inhibitors, but this requires validation [21].

Furthermore, with the entry of PARPi in the treatment armamentarium of mCRPC, in indications that partially overlap with Cabazitaxel, but also of other drugs such as LuPSMA, it becomes more and more important to assess the activity of all these drugs in different DDRs subtypes and their best sequencing. The current literature, with predominantly retrospective data, suggests that prior exposure to PARP inhibitors may negatively impact the therapeutic efficacy of LuPSMA radioligand therapy in DDR-mutated patients [22]. Raychaudhuri et al. observed a reduction in overall survival (OS) and a lack of prostate-specific antigen (PSA) response, particularly in BRCA2-mutated patients receiving sequential treatment with PARP inhibitors followed by LuPSMA. While BRCA2 alterations are well-recognized, independent adverse prognostic factors, these findings underscore the critical need for prospective clinical trials. The ongoing LU-PARP trial is evaluating the efficacy and safety of combined LuPSMA and PARP inhibitor therapy, with preliminary results demonstrating encouraging efficacy and tolerability profiles [23].

The strategy of combining PARP inhibitors with other active agents for prostate cancer has also been explored in relation to their combination with NHT. Preclinical data suggested a potential synergy between PARP inhibitors and NHT, based on the premise that PARP-1 is a potent modulator of androgen receptor (AR) function, regulating its association with chromatin. Consequently, PARPi sensitizes prostate cancer cells to both genotoxic insults and androgen deprivation [24]. TALAPRO2, MAGNITUDE, and PROPEL have explored this issue, combining Enzalutamide and Talazoparib and Abiraterone and Niraparib/Olaparib, respectively. Collectively, the results from clinical trials and multiple meta-analyses confirm that the combination of PARP inhibitors (PARPi) and NHT significantly prolongs radiographic progression-free survival (rPFS) in patients with metastatic castration-resistant prostate cancer (mCRPC) harboring BRCA1/2 and homologous recombination repair (HRR) gene mutations, regardless of the specific PARPi agent utilized [25,26,27]. This is of critical relevance given that clinically meaningful responses to PARPi monotherapy have been largely confined to BRCA1/2-mutant mCRPC cohorts, thereby highlighting the mechanistic synergy and enhanced therapeutic efficacy achieved through PARPi and NHT co-administration [24]. This benefit appears to be evident even in the early stages of the disease, as demonstrated by the AMPLITUDE trial that met its primary endpoint of improved rPFS, demonstrating the efficacy of combining a PARPi (niraparib) with NHT in mCSPC patients with DDR alterations [28]. Patients with BRCA variants derived the greatest benefit from the combination. Improvements in rPFS were accompanied by a statistically significant delay in time to symptomatic progression and a trend toward improved overall survival [28]. As a result, PARP inhibitor-based combinations are increasingly incorporated into first-line treatment recommendations in mCRPC according to recent ESMO and Italian guidelines [9,11]. Furthermore, all of these data strengthen the concept that DDR alteration represents solid actionable therapeutic targets rather than solely prognostic biomarkers [29]. Moreover, given the recent reshape of therapeutic landscape, with the possibility of combined PARPinhibitors and NHT even in the castration-sensitive setting, but also considering the possibility of triplet therapy with Docetaxel in mCSPC, there is an emerging challenge about the optimization of treatment sequencing for DDR-mutated patients. In particular for BRCA1/2-mutated high-volume mCPSC patients, there is no direct comparative evidence that currently support one strategy over another: Docetaxel-based triplet therapy has demonstrated the most mature OS data in unselected high-volume disease [30,31], while PARP inhibitor-based intensification has shown substantial rPFS benefit in BRCA-altered tumors [25,26,27,28]. While, in the literature, there is no data about ideal treatment sequence for this patients [9], it has already been highlighted that the mutational information about DDR alterations is needed as soon as possible [9,11]: as a consequence, FFPE analysis should be carried out as soon as the patient is diagnosed with mCSPC, with possible integration with liquid biopsy procedure, in order to overcome both FFPE and liquid biopsies limitations [11,12,14]. In this context, our exploratory findings regarding differential outcomes with taxane- and ARSI-based therapies may contribute to hypothesis generation for future biomarker-driven sequencing studies. Finally, these results remain directly applicable in oncological centers in which liquid biopsy is still not easily accessible, and in patients who have not received upfront PARP inhibitor-based combinations or triplet therapy, a clinical scenario that continues to be encountered in routine practice. Furthermore, our data provide historical real-world evidence that may serve as a reference for evaluating treatment sequencing in the evolving era of biomarker-driven prostate cancer management.

## 5. Conclusions

In conclusion, our study confirms that one-fourth of metastatic prostate cancer patients harbor variants of DDR genes. Positive family history is a strong predictor of mutated DDR status. BRCA1/2 alterations were associated with shorter duration of the castrate-sensitive phase. With the limitation of the heterogeneity of the real-world population, DDRmut and DDRwt cohorts showed similar clinical and pathological characteristics, and similar PFS and OS across various lines of treatments. Although our findings suggest a potential signal favoring first-line taxane chemotherapy in BRCA1/2–ATM-mutated patients, this observation should be interpreted cautiously due to the limited sample size and the retrospective nature of the study. These results are only hypothesis-generating and require validation in larger prospective studies; still, our data reinforce the idea that BRCA1/2–ATM-mutated patients benefit from standard therapies, and that the predictive impact of the other DDR alterations varies according to the type of treatment and gene concerned.

## Figures and Tables

**Table 1:** Main clinical and pathological characteristics of DDRmut and DDRwt patients.

	DDRwt	DDRmut	ATM/BRCA1/BRCA2	*p*-Value
Patients	225	63	47	
Median age at diagnosis (years)	65.00 [60.00–71.00]	67.00 [58.00–72.00]	66.00 [59–72]	0.8613
Gleason Score (GS)				0.3348
6–7	50	13	10	
8–10	175	49	35	
Median PSA at diagnosis (IQR)	18.00 [9.00–70.00]	22.40 [8.05–88.25]	21 [9.70–95.00]	0.6833
Neuroendocrine features at histology	2	2	0	0.4366
M1 at diagnosis	107	32	29	0.6671
mCRPC at the time of analysis	211	46	26	0.89

**Table 2:** Familiar history for cancer in DDRwt and DDRmut patients.

Family History of Cancer	DDRwt	DDRmut	ATM/BRCA1/BRCA2	OR (95% CI)	*p*-Value
Positive for any of the typical BRCA-related tumors	35	31	30	5.42 (2.9–10.1)	<0.0001
Ovary	22	24	18	5.83 (2.9–11.5)	<0.0001
Breast	6	3	3	1.84 (0.45–7.45)	0.6474
Pancreas	5	0	0	NA	0.5248
Prostate	7	6	9	3.35 (1.05–10.7)	0.0634

**Table 3:** Median duration of hormone-sensitive phase between DDRmut, ATM/BRCA1–2 mut, and DDRwt patients.

DDR Mutational Status	Patients (N)	Median (Months)	Quartiles	Range	*p*-Value (Log Rank Test)
DDRwt (+VUS)	211	44	14.00–70.00	1–227	0.20
DDRmut (all variants except VUS)	46	36	11.00–54.75	5–164	0.81
ATM/BRCA1–2 mut (except VUS)	26	25	8.00–63.10	2–170	0.003

**Table 4:** Multivariable Cox model for PFS and OS for first-line mCRPC treatment.

Endpoint	Variable	HR (95% CI)	*p*-Value
First-line mCRPC PFS	DDR-mutated status (vs. DDRwt)	1.01 (0.69–1.47)	0.968
	Age at diagnosis	1.07 (0.87–1.32)	0.516
	Gleason score ≥8 (vs. <8)	1.18 (0.80–1.74)	0.418
	Metastatic disease at diagnosis (M1 vs. M0)	1.28 (0.81–2.02)	0.291
	High-volume disease (CHAARTED)	0.69 (0.37–1.30)	0.252
	Prior systemic intensification in mHSPC beyond ADT	1.89 (1.15–3.12)	0.013
	First-line taxane (vs. ARSI)	2.13 (1.45–3.15)	<0.001
First-line mCRPC OS	DDR-mutated status (vs. DDRwt)	1.25 (0.67–2.33)	0.484
	Age at diagnosis	1.47 (0.99–2.17)	0.055
	Gleason score ≥8 (vs. <8)	1.72 (0.91–3.24)	0.095
	Metastatic disease at diagnosis (M1 vs. M0)	1.33 (0.57–3.10)	0.507
	High-volume disease (CHAARTED)	0.37 (0.12–1.15)	0.085
	Prior systemic intensification in mHSPC beyond ADT	3.74 (1.63–8.55)	0.002
	First-line taxane (vs. ARSI/)	0.29 (0.12–0.69)	0.005

**Table 5:** Comparative treatment outcomes according to DDR mutational status. NHT includes abiraterone and enzalutamide; first-line taxane includes docetaxel or Cabazitaxel-based regimens.

Treatment Setting	Outcome	DDRwt Median Months (95% CI)	DDRmut Median Months (95% CI)	HR (95% CI)	Log Rank *p*
First-line NHT	PFS	18 (12–25)	17 (4–17)	0.88 (0.49–1.20)	0.057
First-line NHT	OS	44 (34–65)	40 (36–57)	0.97 (0.24–1.016)	0.305
First-line taxane	PFS	10 (7–12)	9.5 (3–34)	1.79 (0.41–1.29)	0.109
First-line taxane	OS	36 (31–68)	39 (20–71)	0.84 (0.41–1.29)	0.114
Cabazitaxel (third-line mCRPC)	PFS	6 (4–7)	7 (3.0–10)	0.99 (0.72–1.03)	0.395
Cabazitaxel (third-line mCRPC)	OS	17 (14–27)	29.5 (24–35)	0.94 (0.53–1.11)	0.282

**Table 6:** Comparative treatment outcomes in BRCA1/2 and ATM mut patients. NHT includes abiraterone and enzalutamide; first-line taxane includes docetaxel or Cabazitaxel-based regimens.

Treatment Setting	Outcome	DDRwt Median Months (95% CI)	BRCA1/2–ATM Median Months (95% CI)	HR (95% CI)	Log Rank *p*
First-line NHT	PFS	18 (12–25)	12 (4.0–17.0)	0.74 (0.35–1.10)	0.048
First-line NHT	OS	44 (54–65)	38 (32.0–41)	0.98 (0.38–1.05)	0.35
First-line taxane	PFS	10 (7.0–12.0)	12.0 (2.0–34.0)	0.88 (041–1.04)	0.274
First-line taxane	OS	36 (31–68)	70 (24–75)	0.64 (0.14–1.91)	0.318
Cabazitaxel (third-line mCRPC)	PFS	6 (4–7)	6 (3–7)	0.86 (0.65–1.20)	0.80
Cabazitaxel (third-line mCRPC)	OS	17 (14–17)	31.3 (24–41)	0.99 (0.69–1.09)	0.582

## Data Availability

The original contributions presented in this study are included in the article. Further inquiries can be directed to the corresponding author(s).

## Abbreviations

The following abbreviations are used in this manuscript:

BRCA	BReast CAncer Gene
ATM	Ataxia-Telangiectasia Mutated Gene
CHEK2	Checkpoint Kinase 2 Gene
FANCA	Fanconi Anemia Gene
CDK12	Cyclin-Dependent Kinase 12 Gene
PALB2	Partner and Localizer of BRCA2 Gene
BRIP1	BRCA1-Interacting Protein 1 Gene
NBN	Nibrin Gene
NHT	Novel Hormonal Therapy
MCRPC	Metastatic Castration-Resistant Prostate Cancer
PARPinh	Poly (ADP-Ribose) Polymerase Inhibitors
DDRwt	DNA Damage Repair Wild Type
DDRmut	DNA Damage Repair Mutated
VUS	Variants of Unknown Significance
PFS	Progression-Free Survival
OS	Overall Survival
ctDNA	Circulating Tumor DNA
cfDNA	Circulating Free DNA
FFPE	Formalin-Fixed Paraffin-Embedded

## Appendix A

**Table A1:** Complete list of mutations.

Gene	Variant	Germline	Mut. Type	VUS	Other Alterations	Kit
BRCA1	p.L1764fs*1	yes	Frameshift			Foundation One
	p.E230fs*3		Frameshift			Foundation One
	p.Q563*		Stop gain			Foundation One
	p.K944*	yes	Stop Gain			Foundation One
BRCA2	p.N969fs*31	yes	Frameshift			Foundation One
	p.C1875_G1908del	yes	Deletion			Foundation One
	p.K343fs*6		Frameshift			Foundation One
	p.S636*		Stop gain			Foundation One
	p.I332fs + p.K437fs		Frameshift			Devyser
	loss		Other (loss)			Foundation One
	p.S2178Y		Missense	Yes (cl 3)		Foundation One
	p.N1287fs*2 + p.S489fs*25	Yes	Frameshift			Foundation One
	p.L1908Rfs*2	Yes	Frameshift			Devyser
	p.W1692fs*3	Yes	Frameshift			Foundation One
	p.V220Ifs*4	Yes	Frameshift			Foundation One
	p.Y2215Lfs*10	Yes	Frameshift			Foundation One
	Loss	Yes	Other (loss)			Foundation One
	p.T3033fs*29	Yes	Frameshift			Foundation One
	p.E514fs*13		Frameshift			Foundation One
	p.G98V		Missense			Foundation One
	p.E462*		Stop gain		PTEN p.K102fs*11 (Frameshift)	Foundation One
	Loss		Other (loss)			Foundation One
	p.E2261K		Missense			Foundation One
	p.Q1107*		Stop gain		PIK3CA p.K944* (Stop gain)	Foundation One
	p.S710*		Stop gain			Foundation One
	p.G2063*	Yes	Stop gain			Foundation One
	p.S1951fs*11	Yes	Frameshift			Foundation One
	p.E1016*		Stop gain			Foundation One
	p.N986fs*2		Frameshift			Foundation One
	p.K943Sfs*8	Yes	Frameshift			Devyser
	p.Q2157fs*18		Frameshift		TP53 p.M160fs*10 (Frameshift)	Foundation One
	loss	Yes	Other (loss)		ATM p.S207C (Missense)	Foundation One
	loss	Yes	Other (loss)			Foundation One
	p.V1804fs*2	Yes	Frameshift			Foundation One
	p.Q3078*		Stop gain	Yes (cl3)		Devyser
	p.N986fs		Frameshift			Devyser
	p.D2071H		Missense	Yes (cl3)		Foundation One
	p.R2494*		Stop gain		ATM p.F2219fs*12 (Frameshift)	Foundation One
	p.Y2905*		Stop gain			Devyser
	p.P1842fs*5		Frameshift			Foundation One
ATM	p.N2241fs*8		Splice donor site variant			Foundation One
	c.7629 + 1G > A	Yes	Stop gain			Foundation One
	p.F2219fs*12	Yes	Frameshift		BRCA2 p.R2494* (stop gain)	Foundation One
	p.Y1961*	Yes	Stop gain			Foundation One
	p.Q1015*		Stop gain			Foundation One
	Loss (biallelic)	Yes	Other (loss)			Foundation One
	p.R2443*		Stop gain			Foundation One
	p.Q161fs*23		Frameshift			Foundation One
	p.S207C		Missense		BRCA2 loss	Foundation One
CHEK2	p.G328fs*10		Frameshift			Foundation One
	p.L174F		Missense	Yes (cl2)	TMPRSS2-ERG fusion	Foundation One
	p.T367fs*15		Frameshift			Foundation One
	p.S434* + p.R221fs*117		Stop gain + frameshift			Foundation One
	p.D438Y		Missense			Foundation One
	p.I157T		Missense			Foundation One
FANCA	p.Q1144*		Stop gain	Yes (cl2)	PTEN loss	Foundation One
	p.T291A		Missense	Yes (cl2)		Foundation One
CDK12	p.S343fs*8 + p.A744fs*9		Frameshift			Foundation One
	p.S377*		Stop gain			Foundation One
PALB2	c.3113 + 2T > G	Yes	Splice site			Foundation One
	p.K628fs*5		Frameshift			Foundation One
	c.2250 + 1G > A		Splice site			Foundation One
BRIP1A	p.P47A		Missense		DNMT3A E477fs*174 (Frameshift)	Foundation One
NBN	p.K219fs*16		Frameshift			Foundation One
